# The Role of Rehabilitation in Arterial Function Properties of Convalescent COVID-19 Patients

**DOI:** 10.3390/jcm12062233

**Published:** 2023-03-13

**Authors:** Maria Ioanna Gounaridi, Angelos Vontetsianos, Evangelos Oikonomou, Panagiotis Theofilis, Nikolaos Chynkiamis, Stamatios Lampsas, Artemis Anastasiou, Georgios Angelos Papamikroulis, Efstratios Katsianos, Konstantinos Kalogeras, Theodoros Pesiridis, Aikaterini Tsatsaragkou, Manolis Vavuranakis, Nikolaos Koulouris, Gerasimos Siasos

**Affiliations:** 1 3rd Department of Cardiology, Sotiria Chest Disease Hospital, Medical School, National and Kapodistrian University of Athens, 11527 Athens, Greece; 2 Rehabilitation Unit, 1st Respiratory Medicine Department, Sotiria Hospital, National and Kapodistrian University of Athens, 11527 Athens, Greece; 3 1st Department of Cardiology, Hippokration General Hospital, Medical School, National and Kapodistrian University of Athens, 11527 Athens, Greece; 4 Cardiovascular Division, Brigham and Women’s Hospital, Harvard Medical School, Boston, MA 02115, USA

**Keywords:** COVID-19, SARS-CoV-2, endothelium-dependent vasodilation, arterial stiffness, flow-mediated dilation, rehabilitation, pulse-wave velocity, cardiovascular disease, long COVID-19 syndrome, post-COVID-19

## Abstract

Coronavirus disease (COVID-19) is a respiratory disease, although arterial function involvement has been documented. We assess the impact of a post-acute COVID-19 rehabilitation program on endothelium-dependent vasodilation and arterial wall properties. We enrolled 60 convalescent patients from COVID-19 and one-month post-acute disease, who were randomized at a 1:1 ratio in a 3-month cardiopulmonary rehabilitation program (study group) or not (control group). Endothelium-dependent vasodilation was evaluated by flow-mediated dilation (FMD), and arterial wall properties were evaluated by carotid–femoral pulse wave velocity (cf-PWV) and augmentation index (AIx) at 1 month and at 4 months post-acute disease. FMD was significantly improved in both the study (6.2 ± 1.8% vs. 8.6 ± 2.4%, *p* < 0.001) and control groups (5.9 ± 2.2% vs. 6.6 ± 1.8%, *p* = 0.009), but the improvement was significantly higher in the study group (rehabilitation) (*p* < 0.001). PWV was improved in the study group (8.2 ± 1.3 m/s vs. 6.6 ± 1.0 m/s, *p* < 0.001) but not in the control group (8.9 ± 1.8 m/s vs. 8.8 ± 1.9 m/s, *p* = 0.74). Similarly, AIx was improved in the study group (25.9 ± 9.8% vs. 21.1 ± 9.3%, *p* < 0.001) but not in the control group (27.6 ± 9.2% vs. 26.2 ± 9.8 m/s, *p* = 0.15). Convalescent COVID-19 subjects of the study group (rehabilitation) with increased serum levels of circulating IL-6 had a greater reduction in FMD. Conclusively, a 3-month cardiopulmonary post-acute COVID-19 rehabilitation program improves recovery of endothelium-dependent vasodilation and arteriosclerosis.

## 1. Introduction

The interaction between coronavirus disease (COVID-19) and the cardiovascular system is multifaceted and well documented, extending from direct or inflammatory injury to the myocardium to endothelial involvement and impairment [1,2,3]. Furthermore, microvascular dysfunction, atherosclerotic plaque progression and vulnerability as well as accelerated arteriosclerosis have been reported [2,4]. Several mechanisms have been implicated in this interplay between COVID-19 and arterial health, including the COVID-19-related inflammatory cascade, the interaction between the severe acute respiratory syndrome coronavirus-2 (SARS-CoV-2) spike protein and angiotensin-converting enzyme 2 (ACE2), and the subsequently increased activity of angiotensin (Ang) II/Ang II receptor type 1 (AT1) [1,5,6].

Importantly, the effects of COVID-19 may extend beyond the acute phase of the disease with symptoms involving multiple organs or systems, and this situation describes post-COVID-19 syndrome [2,7,8]. Vascular function and atherosclerosis data show that up to 6 months post-acute COVID-19, subjects continue to present impairment of vascular function parameters, which may be implicated in a situation of accelerated atherosclerosis [9].

The multi-organ nature of post-COVID-19 syndrome requires multi-disciplinary attention [10]. Cardiopulmonary rehabilitation has been advocated to improve the functional status of patients with respiratory and cardiovascular diseases [11,12]. Moreover, rehabilitation in patients with chronic pulmonary disease may improve vascular function parameters [13]. Additionally, scarce evidence in post-COVID-19 patients suggests that endothelium-dependent vasodilation may be ameliorated after cardiopulmonary rehabilitation [14,15]. Therefore, we hypothesize that cardiopulmonary rehabilitation exerts beneficial effects in post-COVID-19 patients via improvement in arterial function properties. Accordingly, we evaluated the impact of a structured cardiopulmonary rehabilitation program on endothelium-dependent vasodilation and arterial wall properties of convalescent subjects from COVID-19 infection. 

## 2. Materials and Methods

### 2.1. Study Population

In this study, we prospectively enrolled 60 adult convalescent patients from COVID-19 infection one month post-acute disease. All patients were enrolled from January to September 2022 when the dominant COVID-19 variable in Greece was omicron (variant B.1.1.529). From the outpatient center, Cardiology Department of Preventive Cardiology, 60 subjects were enrolled and served as the control group. Convalescent COVID-19 subjects were randomized at a 1:1 ratio to participate or not in a rehabilitation program. From the study, we excluded patients (a) with end-stage renal disease; (b) with active malignancy; (c) recently hospitalized with another infection; (d) with active COVID-19 infection; and (e) unable/not willing to consent to participate. Individuals were evaluated monthly for COVID-19 re-infection via nasopharyngeal PCR swabs, with those testing positive being further excluded from the study.

For the convalescent COVID-19 patients, all arterial functional parameters were evaluated at two times points. The first time (T0) was at one month following recovery from acute COVID-19, and the second time (T1) was 3 months later. During each study visit, all measurements were performed in the same order with blood samples drawn at the end of the study enrollment visit. 

The hospital’s ethics committee with collaboration from the Athens Medical School of the National and Kapodistrian University of Athens, Greece, approved the study (protocol number: 23464/07-09-20), which was carried out following the Declaration of Helsinki (1989). All subjects provided written consent after being thoroughly informed about the study’s aims and procedures. 

### 2.2. Rehabilitation Program

Patients in the rehabilitation group entered the program 1 month following recovery from acute COVID-19. All subjects randomized to the rehabilitation program participated in 8 outpatient rehabilitation sessions for a total of 1 month, consisting of 30 min interval aerobic exercise on a stationary bike at 100% of peak work rate along with resistance exercises for the upper and lower limbs. Then, for the next 2 months, they followed a remote home-based program with 24 sessions consisting of 30 min walking with an individualized target of steps (recorded via a mobile app installed in the patient’s mobile phone) along with a specific set of resistance exercises for the upper and lower limbs using elastic bands. The assessors set new step targets based on the steps and the symptoms reported by the patient. If dyspnea and leg discomfort were both <4 on the Borg scale [16], the weekly target of steps was increased by 5–10%, otherwise the target remained the same. Patients in the no rehabilitation group were advised to be physically active.

### 2.3. Flow-Mediated Dilation

Flow-mediated dilation (FMD) in the brachial artery was used to evaluate endothelium-dependent vasodilation [17,18,19] at enrollment (T0) and three months later (T1). The FMD technique examines the endothelium-dependent dilation of the peripheral artery. This method was originally introduced in 1992 and essentially evaluates the production of nitric oxide (NO) by the endothelium. For the examination, the patient stays in supine position for 10–15 min, and the right brachial artery is located through Doppler ultrasound 5 cm above the antecubital fossa. In this study, the Vivid-e Ultrasound system (General Electric, Milwaukee, WI, USA) was used, with a 5.0–13.0 MHz (harmonics) linear array ultrasound transducer. After the brachial artery is located, its diameter is measured longitudinally at various points, and the average of the multiple measured diameters is calculated. Then, a cuff with an air chamber is placed on the forearm at a pressure 50 mmHg higher than the patients systolic blood pressure in order to induce ischemia. After 5 min, the cuff is released, resulting in hyperemia to the forearm and dilation of the brachial artery. During the hyperemia the brachial artery, the diameter is measured again every 15 s for 2 min, and the average is derived from multiple measurements along the vessel. The diameter is measured during the same phase of the cardiac cycle each time, as it is synchronized with ECG during the examination. All diameters were measured at the boundary of the media–adventitia interface. FMD was then calculated as the percent change of vessel diameter from rest to the maximum diameter after cuff release. All tests were performed by the same operator throughout the study. 

### 2.4. Pulse Wave Velocity and Augmentation Index

We evaluated arterial wall properties by measurement of pulse wave velocity (PWV) and augmentation index (AIx) at enrollment (T0) and three months later (T1). In this study, the SphygmoCor device (AtCor Medical) was used to measure carotid femoral pulse wave velocity (cf-PWV). PWV reflects the speed in which the arterial pressure waves travel along the aorta and great arteries. It can be calculated by dividing the distance between the two arterial recording points by the travel time of the pressure waves between them. Cf-PWV is generally accepted as the “gold standard” measurement in the evaluation of arterial stiffness and can be used to predict cardiovascular events. The measurement is usually achieved with a set of devices that can measure PWV and can also analyze the pulse pressure waveform [20,21,22,23].

In this study, all subjects were placed in a quiet room, with no temperature fluctuation, and they rested supine for at least 10 min. All measurements were performed at the right common carotid and femoral arteries. Patients were advised not to speak during measurements. The distance between the suprasternal notch and the femoral artery was measured in a straight line using a measuring tape as well as the distance from the carotid artery to the suprasternal notch, and then, the difference between the two was calculated [21]. The value of PWV was derived from the pulse transit time and the distance measured between the two recording sites. A simultaneous ECG was recorded in order to synchronize the pressure waves. All tests were performed by the same expert operator. Augmentation index (AIx) of the central (aortic) pressure waveform was calculated to evaluate wave reflections. A validated acquisition system (SphygmoCor, AtCor Medical, Sydney, NSW, Australia) was used to noninvasively capture and analyze arterial pulse through applanation tonometry. Higher values of AIx correlate with increased arterial stiffness. With increasing stiffness, there is faster propagation of the forward pulse wave as well as a faster reflected wave. This results in the reflected wave reaching the central aorta earlier and increases the pressure in late systole. Accordingly, the augmentation index = (amplified pressure/pulse pressure) rises. A correction of AIx based on a heart rate of 75 bpm is needed, as AIx can be influenced by the heart rate. Radial pressure is calibrated using the systolic and diastolic blood pressure measured with a sphygmomanometer in the brachial artery [24,25,26].

### 2.5. Laboratory Measurements

Blood samples were drawn at study enrollment (T0) for all participants. They were centrifuged and refrigerated at a temperature of −80 °C until assayed. A Luminex assay was used to measure the levels of interleukin-6 (IL-6), a well-established inflammatory cytokine, in patients’ serum. (Thermo Fisher Scientific Inc., Waltham, MA, USA) [27].

### 2.6. Statistical Analysis

Regarding continuous variables, the Kolmogorov–Smirnov test and visual inspection of P-P plots were used to test for normality of distribution. Accordingly, they were presented as mean with standard deviation or median with 25th and 75th quartile (for normally or not normally distributed variables, respectively). Valid frequencies with percentage were used to present categorical variables. Differences in continuous variables across the groups were evaluated with the *t*-test or the Mann–Whitney U test, depending on the normality of their distribution. Differences in categorical variables across the groups were calculated by formation of contingency tables and performance of χ^2^ test. A repeated-measures (paired) *t*-test was executed to determine the overtime changes in vascular function markers in the intervention and the control group of convalescent COVID-19 individuals. To assess the interactions of the overtime changes in the examined variables according to the intervention group, a general linear model was applied. All reported *p* values were based on two-sided hypotheses. When the *p* value was less than 0.05, differences were considered statistically significant. Adherence to the remote home-based exercise rehabilitation program was calculated as the rate of the sessions completed by the patients over the total sessions. All statistical calculations were performed in SPSS software (version 27.0; SPSS Inc., Chicago, IL, USA).

## 3. Results

### 3.1. Baseline Characteristics of the Study Population

The entire study population consisted of 60 convalescent COVID-19 individuals and 60 healthy controls (Supplementary Table S1). There were no major differences in age, sex, or the prevalence of major cardiovascular risk factors across the two groups. The adherence rate to the remote home-based rehabilitation program was 85%. The main reasons that patients missed rehabilitation sessions were weather and health issues (pain, feeling unwell). The inflammatory burden, as assessed by circulating IL-6 levels, was similar in the two groups (convalescent COVID-19: 1.74 (0.64, 4.97) pg/mL vs. control: 1.31 (0.98, 2.70) pg/mL, *p* = 0.95). However, convalescent COVID-19 patients had impaired vascular function, as evidenced by reduced FMD (convalescent COVID-19: 6.1 (1.9)% vs. control: 7.4 (3.2)%, *p* = 0.02), increased PWV (convalescent COVID-19: 8.4 (1.6) m/s vs. control: 7.3 (0.8) m/s, *p* < 0.001) and increased AIx (convalescent COVID-19: 26.5 (9.5)% vs. control: 23.4 (8.5)%, *p* < 0.08).

The baseline characteristics of the convalescent COVID-19 patients according to the performance of rehabilitation are presented in Table 1. The age and sex distribution were balanced across the two groups. Moreover, we did not observe any statistically significant differences concerning the presence of cardiovascular risk factors (BMI, history of DM, arterial hypertension, and dyslipidemia). Regarding the disease course in the acute phase, the individuals were evenly distributed. We did not detect considerable differences in the examined vascular function markers, namely FMD (rehabilitation: 6.2 (1.8)% vs. no rehabilitation: 5.9 (2.2)%, *p* = 0.57), PWV (rehabilitation: 8.2 (1.4) m/s vs. no rehabilitation: 8.8 (1.9) m/s, *p* = 0.10), and AIx (rehabilitation: 25.9 (9.8)% vs. no rehabilitation: 27.6 (9.2), *p* = 0.28). Circulating IL-6 levels did not differ significantly between the two groups (rehabilitation: 1.74 (0.64, 6.46) pg/mL vs. no rehabilitation: 2.03 (0.95, 2.98) pg/mL, *p* = 0.77).

### 3.2. The Impact of Rehabilitation on Vascular Function Recovery Post-COVID-19

**Figure 1:**
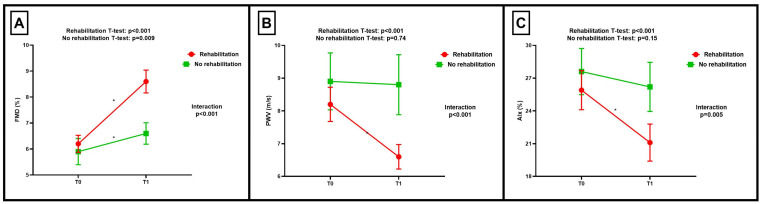
Over-time changes in vascular function markers according to the performance of rehabilitation. (**A**) Superimposed symbols with connecting line plot showing the change in flow-mediated dilation (FMD) from baseline to 1 month post-acute COVID-19 (T0) and to 4 months post-acute COVID-19 (T1) according to the performance of rehabilitation. (**B**) Superimposed symbols with connecting line plot showing the change in pulse wave velocity (PWV) from baseline to 1 month post-acute COVID-19 (T0) and to 4 months post-acute COVID-19 (T1) according to the performance of rehabilitation. (**C**) Superimposed symbols with connecting line plot showing the change in augmentation index (AIx) from baseline to 1 month post-acute COVID-19 (T0) and to 4 months post-acute COVID-19 (T1) according to the performance of rehabilitation. * Indicates statistically significant difference between T0 and T1.

The influence of rehabilitation on vascular function recovery is illustrated in Table 2. Both groups exhibited statistically significant improvements in FMD across the recovery period (Figure 1A). However, rehabilitation resulted in improvements of greater magnitude when compared to no rehabilitation (*p* for interaction <0.001). Regarding PWV, its remarkable lowering was only observed in the rehabilitation group, while it remained practically unchanged in the control group (T0: 8.9 (1.8) m/s vs. T1: 8.8 (1.9) m/s, *p* = 0.74) (Figure 1B). A significant interaction of PWV with the intervention was noted (*p* < 0.001). Finally, similar findings were seen with AIx, which was significantly diminished across the follow-up only in individuals undergoing rehabilitation (T0: 25.9 (9.8)% vs. T1: 21.1 (9.3), *p* < 0.001) (Figure 1C). Accordingly, we also confirmed a significant interaction of AIx with the intervention (*p* = 0.005).

### 3.3. Interleukin-6 and the Effect of Rehabilitation on Endothelium-Dependent Vasodilation in Post-COVID-19

**Figure 2:**
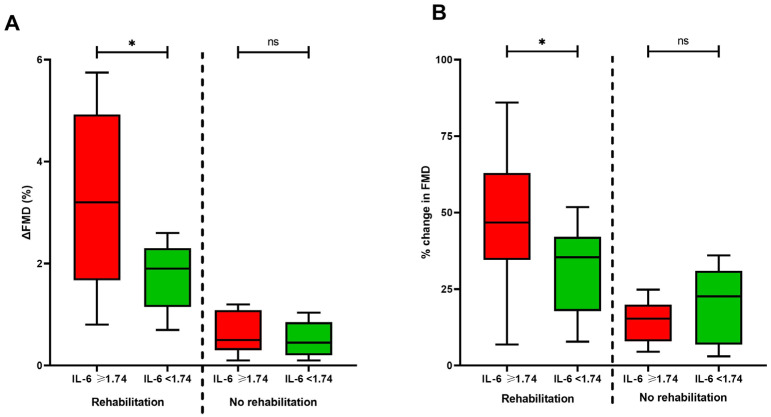
Differences in the change of FMD according to baseline median circulating IL-6 levels of individuals who underwent rehabilitation or not. (**A**) Box plots showing the absolute change in FMD (ΔFMD) in individuals with baseline circulating IL-6 levels above or below the median (1.74 pg/mL). (**B**) Box plots showing the percentage change in FMD in individuals with baseline circulating IL-6 levels above or below the median (1.74 pg/mL). * Indicates statistically significant difference between T0 and T1. ns: non-significant. FMD: Flow-mediated dilation; IL-6: Interleukin 6.

We also assessed whether the inflammatory burden post-COVID-19 could be a determinant of rehabilitation effectiveness. To begin with, we stratified subjects that underwent rehabilitation according to the median circulating IL-6 concentration (1.74 pg/mL). As shown in Figure 2, we observed that individuals presenting with baseline IL-6 concentration above the median had a greater reduction in FMD (ΔFMD) (IL-6 ≥ 1.74 pg/mL: 3.18 (1.78)% vs. IL-6 < 1.74 pg/mL: 1.74 (0.68)%, *p* = 0.007), as well as percentage change in FMD (IL-6 ≥ 1.74 pg/mL: 57.0 (46.4)% vs. IL-6 < 1.74 pg/mL: 30.8 (13.8)%, *p* = 0.04). Convalescent COVID-19 patients who did not undergo rehabilitation did not exhibit significant changes in ΔFMD (IL-6 ≥ 1.74 pg/mL: 0.70 (0.40)% vs. IL-6 < 1.74 pg/mL: 0.82 (1.65)%, *p* = 0.86) and percentage change in FMD (IL-6 ≥ 1.74 pg/mL: 16.5 (15.2)% vs. IL-6 < 1.74 pg/mL: 23.4 (39.7)%, *p* = 0.68) according to IL-6 levels.

## 4. Discussion

In this study, we confirmed the impaired vascular function properties of convalescent COVID-19 subjects compared to healthy individuals with similar risk factor profiles. Moreover, we noted a gradual recovery of both endothelium-dependent vasodilation and arterial stiffness over time from acute COVID-19. Importantly, we documented that a structured rehabilitation program may accelerate or improve the recovery of arterial function parameters. Additionally, we documented that convalescent COVID-19 subjects with systemic inflammation and increased serum levels of circulating IL-6 may benefit more in terms of endothelium-dependent vasodilation from a rehabilitation program.

In patients with COVID-19, endothelial dysfunction and altered arterial wall properties have been described not only at the acute phase of the disease but also at the recovery phase and even six months post-acute COVID-19 [28,29,30,31,32]. Infection from SARS-CoV-2 may involve many organs and systems, with the inflammatory cascade and endothelial dysfunction as the substrate of several disease manifestations [1].

“Post-acute COVID-19” or ‘’long COVID-19’’ syndrome is characterized by persistent symptoms and/or long-term complications beyond 3 or 4 weeks from the onset of acute symptoms [1,28,33,34]. These patients share common symptoms such as fatigue, post-exertional malaise, muscle weakness, and cognitive dysfunction, impacting everyday functioning and quality of life [35,36]. The underlying mechanisms contributing to the pathophysiology of post-acute COVID-19 have not been fully elucidated but might include persistent immune and inflammatory activation and target organ damage as well as the expected sequelae of post-critical illness and long hospital stays [34].

We have previously reported that patients with long COVID-19 present impaired endothelium-dependent vasodilation compared to their counterparts without long-COVID-19 [9,37]. Endothelium-dependent vasodilation and impaired ventriculoarterial coupling [2] may contribute to the functional limitations of post-COVID-19, including multisystem deconditioning and subsequent exercise intolerance and muscle weakness [38,39].

### 4.1. Arterial Stiffness in Convalescent COVID-19 Subjects

Subjects with COVID-19 present increased arterial stiffness as measured by PWV and AIx compared to matched control subjects without infection from SARS-CoV-2 [2,40,41]. Moreover, increased arterial stiffness may last up to six months post-acute infection and may be associated with sympathetic drive and endothelial dysfunction [2,9]. Endothelial impairment at the post-acute COVID-19 phase may contribute to increased arterial stiffness. The role of systemic inflammation may also affect arterial function parameters [42,43]. Additionally, the role of sympathetic drive has been described in the impaired ventriculoarterial coupling of convalescent COVID-19 subjects [2], which may contribute to long COVID-19 symptoms, especially regarding functional status and fatigue. The sympathetic overdrive was documented in asymptomatic individuals during COVID-19 convalescence, through multiple non-invasive measures (low/high-frequency power ratio, mean standard deviation of normal-to-normal intervals, and root mean square of successive RR interval differences) [44]. The above-mentioned findings may be more pronounced in survivors of severe acute COVID-19 at convalescence [45].

A recently reported study by Zanoli et al. further illustrates the impact of COVID-19 on vascular status [46]. The investigators noted abnormalities in various arterial stiffness measures (aortic PWV, carotid Young’s elastic modulus and distensibility) 12–48 weeks after COVID-19 [46]. The degree of PWV impairment at convalescence was dependent on the inflammatory burden during the acute phase of the disease [46]. Despite improvement in arterial stiffness measures being noted during long-term follow-up, these do not normalize [46]. Future studies should determine the importance of such findings in the long-term cardiovascular prognosis of COVID-19 survivors who present persistently impaired arterial stiffness.

### 4.2. Cardiopulmonary Rehabilitation

Cardiopulmonary rehabilitation has been supported for several decades as a way to provide comprehensive care and to improve the functional status of patients with respiratory and cardiovascular diseases. It helps improve exercise capacity and patient’s quality of life, and it prevents long-term complications [11,47]. Aerobic exercise has been shown to improve endothelial function, both in large arteries and in microcirculation. It can modify the blood flow pattern at arterial branch points, thus leading to less turbulent flow. This reinforces the expression of genes with atheroprotective properties, mainly the NO synthase (eNOS), thus leading to the existence of a vasodilatory and vasoprotective environment in the endothelium. Other vasoprotective factors such as PGI2 and EDHF also appear to increase, which may lead to the improved physiological actions of the endothelium from exercise. Improving autonomic tone and reducing inflammation and oxidative stress may all contribute to the positive effects of exercise on endothelial function and decrease arterial stiffness [12,48,49]. The improvement in endothelial function appears to be largely independent of the type of aerobic exercise, such as treadmill walking versus cycling, as well as continuous versus interval training [50,51]. Moreover, in patients with chronic obstructive pulmonary disease, rehabilitation may improve arterial stiffness parameters (i.e., PWV and AIx) [14,52,53].

Several studies have been published investigating the effects of various exercise programs on reducing symptoms associated with hospitalization following COVID-19 infection, but they have focused on the improvement of patient functional status, quality of life, and respiratory function [54,55,56].

Limited data exist on the role of rehabilitation on endothelial function of post-COVID-19 subjects [15,57]. Similar to our results, significant improvement in clinically evaluated endothelial function after multidisciplinary rehabilitation with a 71% increase in FMD compared to baseline values was documented following rehabilitation; however, this study does not provide evidence on the additional impact of rehabilitation on the natural over-time recovery of arterial function [15].

The mechanisms underlying improvement of endothelial function following cardiac rehabilitation include increased shear stress and activation of endothelial NO synthase [58,59], improved anti-oxidant capacity [60] and regulation of immune system activation [61] and of inflammatory status [62]. Especially, in animal models, aerobic exercise training may modulate IL-6 expression [63]. Our findings show that patients with higher IL-6 levels at baseline present higher improvement of endothelial function following rehabilitation.

In line with the benefits of cardiac rehabilitation, other techniques such as enhanced external counterpulsation may also positively influence the quality of life of affected individuals. As recently shown in a retrospective study, convalescent COVID-19 patients improved several indices associated with quality of life and exercise capacity after 15–35 treatment sessions [64]. It may also enhance cognitive function in affected individuals [65]. An ongoing randomized trial (NCT05668039) on the effectiveness of this method in improving long-COVID-19 fatigue is expected to provide further evidence.

The results of our study should be interpreted with caution, since the relatively small sample size may limit its credibility. Moreover, the menstrual status and the examination of premenopausal female patients during various phases of the menstrual cycle could be confounding factors that were not taken into account. However, it is considered unlikely that this could affect the overall outcome of the study since the number of pre-menopausal women was limited. Additionally, the use of contraception was not recorded. Finally, we solely assessed endothelium-dependent vasodilation in this study with the use of FMD, and the effect of cardiopulmonary rehabilitation in other methods of endothelium-dependent vasodilation, as well as in endothelium-independent vasodilation, is unclear. 

## 5. Conclusions

Convalescent COVID-19 subjects present impaired endothelium-dependent vasodilation and increased arterial stiffness. A 3-month cardiopulmonary rehabilitation program in the post-acute COVID-19 phase improves recovery of endothelium-dependent vasodilation, arterial stiffness, and arterial reflected waves, especially in patients with high levels of IL-6 before the initiation of cardiac rehabilitation. These findings may shed light on the mechanisms underlying the beneficial effects of a cardiopulmonary rehabilitation program and on the selection of subjects with the higher possibility of improvement, in terms of arterial function parameters, following rehabilitation.

## Figures and Tables

**Table 1:** Baseline characteristics of the study population.

	Rehabilitation (*n* = 30)	No Rehabilitation (*n* = 30)	*p*
Age, years	54.0 (11.8)	49.1 (12.7)	0.13
Male sex, %	14 (46.7)	18 (60.0)	0.20
BMI, kg/m^2^	26.8 (4.7)	28.6 (4.9)	0.15
Smoking, %	13 (43)	11 (37)	0.60
Diabetes mellitus, %	4 (13.3)	0	0.10
Hypertension, %	8 (26.7)	5 (16.7)	0.35
Dyslipidemia, %	12 (40.0)	10 (33.3)	0.79
Disease course			
Mild Non-Hospitalized, %	5 (16.7)	10 (33.3)	0.11 *
Moderate Hospitalized, %	18 (60.0)	18 (60.0)	
Severe Hospitalized, %	7 (23.3)	2 (6.7)	
Interleukin-6, pg/mL	1.74 (0.64, 6.46)	2.03 (0.95, 2.98)	0.77
Vascular function			
FMD, %	6.2 (1.8)	5.9 (2.2)	0.57
PWV, m/s	8.2 (1.4)	8.9 (1.8)	0.10
AIx, %	25.9 (9.8)	27.6 (9.2)	0.49

BMI: body mass index, FMD: flow-mediated dilation, PWV: pulse wave velocity, AIx: augmentation index. Continuous variables are presented as mean (standard deviation) or as median (25th, 75th quartile) according to their distribution. Categorical variables are presented as frequency (percentage). The Mann–Whitney U or the *t*-test were used to assess the between-group differences in continuous variables. The χ^2^ test was used to assess the between-group differences in categorical variables. * *p* value is referred to as the distribution of mild non-hospitalized, moderate hospitalized and severe hospitalized COVID-19 across the rehabilitation and the no rehabilitation groups.

**Table 2:** Changes in vascular function markers according to the intervention.

	T0	T1	*p*	P_interaction_
FMD_study_	6.2 (1.8)	8.6 (2.4)	<0.001	<0.001
FMD_control_	5.9 (2.2)	6.6 (1.8)	0.009	
PWV_study_	8.2 (1.4)	6.6 (1.0)	<0.001	<0.001
PWV_control_	8.9 (1.8)	8.8 (1.9)	0.74	
AIx_study_	25.9 (9.8)	21.1 (9.3)	<0.001	0.005
AIx_control_	27.6 (9.2)	26.2 (9.8)	0.15	

FMD: flow-mediated dilation, PWV: pulse wave velocity, AIx: augmentation index. T0: 1-month post-acute COVID-19; T1 4-months post-acute COVID-19. Continuous variables are presented as mean (standard deviation). A paired sample *t*-test was used to assess the over-time changes in the examined variables in each group. A repeated measures analysis of variance was used to determine the interaction between the over-time changes in the examined variables and the intervention.

## Data Availability

The data presented in this study are available on request from the corresponding author.

## Supplementary Materials

The following supporting information can be downloaded at: https://www.mdpi.com/article/10.3390/jcm12062233/s1, Table S1: Baseline characteristics of the study population.
