# Supplementary material for: The Role of Rehabilitation in Arterial Function Properties of Convalescent COVID-19 Patients
Source: J Clin Med. 2023 Mar 13;12(6):2233. doi: 10.3390/jcm12062233 (PMC10056228; doi:10.3390/jcm12062233)
Supplement: Supplementary file 1 [file jcm-12-02233-s001.zip › jcm-2156331-supplementary.pdf]

Journal of
Clinical Medicine

Table S1. Baseline characteristics of the study population.

	Convalescent COVID-19 (n = 60)	Controls (n = 60)	p
Age, years	52.2 (12.3)	55.3 (9.8)	0.14
Male sex, %	29 (48.3)	32 (53.3)	0.58
BMI, kg/m^2	28.5 (3.2)	27.4 (4.8)	0.15
Smoking, %	24 (40)	22 (37)	0.71
Diabetes mellitus, %	4 (6.7)	1 (1.7)	0.18
Hypertension, %	13 (21.7)	19 (20.0)	0.82
Dyslipidemia, %	22 (36.7)	19 (31.7)	0.56
Interleukin-6, pg/ml	1.74 (0.64, 4.97)	1.31 (0.98, 2.70)	0.95
**Vascular function**			
FMD, %	6.1 (1.9)	7.4 (3.2)	0.02
PWV, m/s	8.4 (1.6)	7.3 (0.8)	<0.001
AIx, %	26.5 (9.5)	23.4 (8.5)	0.08

BMI: body mass index, FMD: flow-mediated dilation, PWV: pulse wave velocity, AIx: augmentation index. Continuous variables are presented as mean (standard deviation) or as median (25th, 75th quartile) according to their distribution. Categorical variables are presented as frequency (percentage). The Mann-Whitney U or the *t*-test were used to assess the between group differences in continuous variables. The $\chi 2$ test was used to assess the between group differences in categorical variables.
